# Association Between Systemic Inflammatory Response Biomarkers and Disease Activity in Systemic Lupus Erythematosus: A Multi-Center Retrospective Study

**DOI:** 10.3390/diagnostics16121944

**Published:** 2026-06-22

**Authors:** Tao Ma, Jiale Zhang, Jie Kong, Hua Wei, Huaixia Hu, Yinshan Zang, Hongjun He, Wenwen Wang, Xiaoxiang Chen, Yingying Gao

**Affiliations:** 1 Department of Rheumatology and Immunology, Affiliated Nantong Clinical College of Nantong University, Nantong First People’s Hospital, Nantong 226001, China; m17826152675@163.com (T.M.); zzjlll0304@163.com (J.Z.); 18018310753@163.com (J.K.); www_ntyy@163.com (W.W.); 2 Department of Rheumatology and Immunology, Northern Jiangsu People’s Hospital, Yangzhou 225001, China; yzweihua2018@163.com; 3 Department of Rheumatology and Immunology, Second People’s Hospital of Lianyungang, Lianyungang 222006, China; huhuaixia@163.com; 4 Department of Rheumatology and Immunology, The Affiliated Suqian First People’s Hospital of Nanjing Medical University, Suqian 223600, China; syzang@163.com; 5 Department of Rheumatology and Immunology, Taixing People’s Hospital, 98 Runtai South Road, Taixing 225400, China; jshehj717@sohu.com; 6 Department of Allergy and Immunology, Renji Hospital Affiliated to Shanghai Jiao Tong University School of Medicine, Shanghai 200001, China

**Keywords:** systemic lupus erythematosus, disease activity, systemic inflammation response index (SIRI), monocyte-to-lymphocyte ratio (MLR), neuropsychiatric lupus, biomarkers

## Abstract

**Objective**: To evaluate the association of routine complete blood count (CBC)-derived inflammatory biomarkers, including neutrophil-to-lymphocyte ratio (NLR), monocyte-to-lymphocyte ratio (MLR), platelet-to-lymphocyte ratio (PLR), and systemic inflammation response index (SIRI), with disease activity and exploratory neuropsychiatric risk stratification in patients with systemic lupus erythematosus (SLE). **Methods**: In this multi-center retrospective study, 579 SLE patients and 282 healthy controls (HCs) were recruited from five clinical centers between 2018 and 2025. NLR, MLR, PLR, and SIRI were calculated from routine CBC parameters. Disease activity was assessed using the SLE Disease Activity Index 2000 (SLEDAI-2K), with high activity defined as SLEDAI-2K ≥ 10. The comparison between SLE patients and HCs was performed as an exploratory descriptive analysis to characterize systemic inflammatory profiles, whereas the primary analyses focused on associations with disease activity and NPSLE-related risk stratification. **Results**: SLE patients exhibited significantly higher levels of SIRI, NLR, PLR, and MLR compared to HCs (all *p* < 0.001). In this exploratory comparison, MLR showed the largest area under the curve for distinguishing SLE patients from HCs (AUC: 0.849, Cut-off: 0.263). In regression analyses, MLR, NLR, PLR, and SIRI were positively associated with SLEDAI-2K score. In multivariable linear regression analysis, MLR was associated with a higher SLEDAI-2K score (*B* = 4.600, 95% CI: 2.039–7.160, *p* < 0.001). In patients with available neuropsychiatric data, MLR, NLR, and SIRI were higher in patients with NPSLE than in those with non-NPSLE, whereas PLR showed no significant difference. SIRI showed modest exploratory discriminatory ability for NPSLE and may provide auxiliary information for NPSLE risk stratification (AUC: 0.710, *p* < 0.001, Cut-off: 1.438). **Conclusions**: Routine CBC-derived inflammatory biomarkers, particularly MLR, NLR, and SIRI, are associated with SLE disease activity and may serve as accessible, low-cost adjunctive tools for rapid clinical assessment. SIRI may provide additional auxiliary information for identifying patients at higher risk of neuropsychiatric involvement. However, these biomarkers should be interpreted as complementary screening or risk-stratification tools rather than substitutes for established disease activity indices or organ-specific evaluations. Further prospective studies are warranted to validate their clinical utility.

## 1. Introduction

Systemic lupus erythematosus (SLE) is a chronic, highly heterogeneous autoimmune disease characterized by immune complex deposition and multi-organ involvement, driven by T and B cell dysregulation and the production of diverse autoantibodies [1,2]. Given its unpredictable clinical course, the prognosis of SLE heavily depends on the timely and accurate assessment of disease activity [3]. Currently, the Systemic Lupus Erythematosus Disease Activity Index 2000 (SLEDAI-2K) serves as the gold standard for clinical evaluation [4]. However, calculating the SLEDAI-2K score is time-consuming and relies on clinical and laboratory parameters that may not always be available in real time. This limitation underscores the need for simple, rapid, and cost-effective adjunctive markers for disease activity assessment and risk stratification. Recent evidence highlights the potential of systemic inflammatory response biomarkers derived from routine complete blood counts (CBC), including the neutrophil-to-lymphocyte ratio (NLR), monocyte-to-lymphocyte ratio (MLR), and platelet-to-lymphocyte ratio (PLR) [5]. More recently, the systemic inflammation response index (SIRI), calculated as neutrophil count x monocyte count/lymphocyte count, has emerged as an integrated biomarker reflecting immune-inflammatory balance [6,7]. While these markers have been evaluated in several autoimmune rheumatic diseases, their value as adjunctive indicators of SLE disease activity and exploratory risk-stratification markers for neuropsychiatric SLE (NPSLE) remains insufficiently characterized [8,9,10]. Therefore, this multicenter retrospective study aimed to evaluate the association of CBC-derived inflammatory biomarkers with SLE disease activity and to explore their auxiliary value for NPSLE risk stratification.

## 2. Materials and Methods

### 2.1. Study Participants

In this multi-center retrospective study, a total of 579 SLE patients were recruited between 2018 and 2025 from five clinical centers including Nantong First People’s Hospital, Northern Jiangsu People’s Hospital, Second People’s Hospital of Lianyungang, The Affiliated Suqian First People’s Hospital of Nanjing Medical University and Taixing People’s Hospital. All enrolled patients met the classification criteria for SLE by the 2019 European League Against Rheumatism/American College of Rheumatology (EULAR/ACR) Classification Criteria for SLE. Patients were classified into the neuropsychiatric SLE (NPSLE) group or the non-NPSLE group according to nervous system involvement. NPSLE was defined based on the 1999 ACR nomenclature for neuropsychiatric syndromes, clinical evaluation, and available objective evidence, including neurological examination, cerebrospinal fluid (CSF) analysis, neuroimaging such as MRI, or electrophysiological testing where available. Neuropsychiatric manifestations primarily attributable to metabolic disturbance, systemic infection, treatment-related complications, primary intracranial lesions, or pre-existing psychiatric disorders unrelated to SLE were excluded. Other exclusion criteria were: (a) presence of other autoimmune diseases such as systemic sclerosis, rheumatoid arthritis, primary Sjögren’s syndrome, myasthenia gravis, or mixed connective tissue disease; (b) end-stage renal disease, severe liver disease, or severe cardiovascular/cerebrovascular disease; (c) malignant tumors; (d) repeated antibiotic within the past month; (e) thrombosis within the past month; (f) incomplete clinical data required for CBC-derived indices or SLEDAI-2K calculation; and (g) psoriatic arthritis. Additionally, 282 healthy individuals undergoing physical examinations during the same period were included from health examination records as a control group. This multicenter study complied with the Declaration of Helsinki and was approved by the Ethics Committee of the primary center, the First People’s Hospital of Nantong [Ethics Number: 2021KT180], with informed consent obtained from all participants.

### 2.2. Data Collection

Demographic data, clinical manifestations, laboratory results, and available treatment information were collected from electronic medical records. CBC testing and SLEDAI-2K assessment were performed during the same clinical visit, and only the first eligible visit for each patient was included. The inflammatory biomarkers were calculated as follows: MLR = monocyte count/lymphocyte count; NLR = neutrophil count/lymphocyte count; PLR = platelet count/lymphocyte count; and SIRI = neutrophil count x monocyte count/lymphocyte count. The SLEDAI-2K score was calculated based on clinical manifestations and laboratory findings from the same visit. High disease activity was defined as SLEDAI-2K ≥ 10. Treatment data, including glucocorticoids, hydroxychloroquine, conventional immunosuppressants, biologics, and targeted agents, were available for patients from Center 1 and were analyzed as an available-case sensitivity analysis (Supplementary Tables S2 and S3). Detailed cumulative corticosteroid dose and recent pulse therapy were not uniformly available across all centers and were therefore acknowledged as limitations.

### 2.3. Statistical Analysis

To account for potential inter-center heterogeneity, the study center was included as a categorical fixed-effect covariate in the multivariable regression models. Center 1 was used as the reference category, and dummy variables were generated for the remaining centers. Because the number of participating centers was limited and the sample size was uneven across centers, fixed-effect adjustment was selected rather than a random-effects model. Four separate models were constructed for MLR, NLR, PLR, and SIRI to avoid multicollinearity caused by overlapping blood cell components. Continuous SLEDAI-2K score was analyzed using multivariable linear regression. Because the SLEDAI-2K distribution was skewed, high disease activity (SLEDAI-2K ≥ 10) was additionally analyzed as a binary outcome using multivariable logistic regression; the logit link function was used only for these binary logistic regression models. To evaluate whether associations were driven by hematological components embedded in SLEDAI-2K, a non-hematologic SLEDAI-2K score was calculated by subtracting leukopenia and thrombocytopenia items from the total SLEDAI-2K score. Covariates were selected based on clinical relevance and included age, sex, disease duration, hemoglobin, ESR, CRP, and study center. Multicollinearity was assessed using variance inflation factors (VIFs), and no severe multicollinearity was observed (all VIFs < 5). The primary multivariable analyses were performed using complete-case analysis. Treatment variables available from Center 1 were summarized and explored in available-case sensitivity analyses. Normally distributed continuous variables were expressed as mean ± standard deviation (SD) and compared using unpaired t-tests or one-way analysis of variance (ANOVA). Non-normally distributed variables were expressed as median (interquartile range [IQR]) and compared using the Mann–Whitney U test or Kruskal–Wallis test. Categorical variables were expressed as *n*/*N* (%) and compared using chi-square tests or Fisher’s exact tests as appropriate. Correlations were evaluated by Spearman correlation analysis. Receiver operating characteristic (ROC) curve analysis was performed for exploratory discriminatory analyses. *p* < 0.05 was considered statistically significant.

## 3. Results

### 3.1. Baseline Characteristics and Comparison of Systemic Inflammation Response Biomarkers Between SLE Patients and Healthy Controls

**Figure 1:**
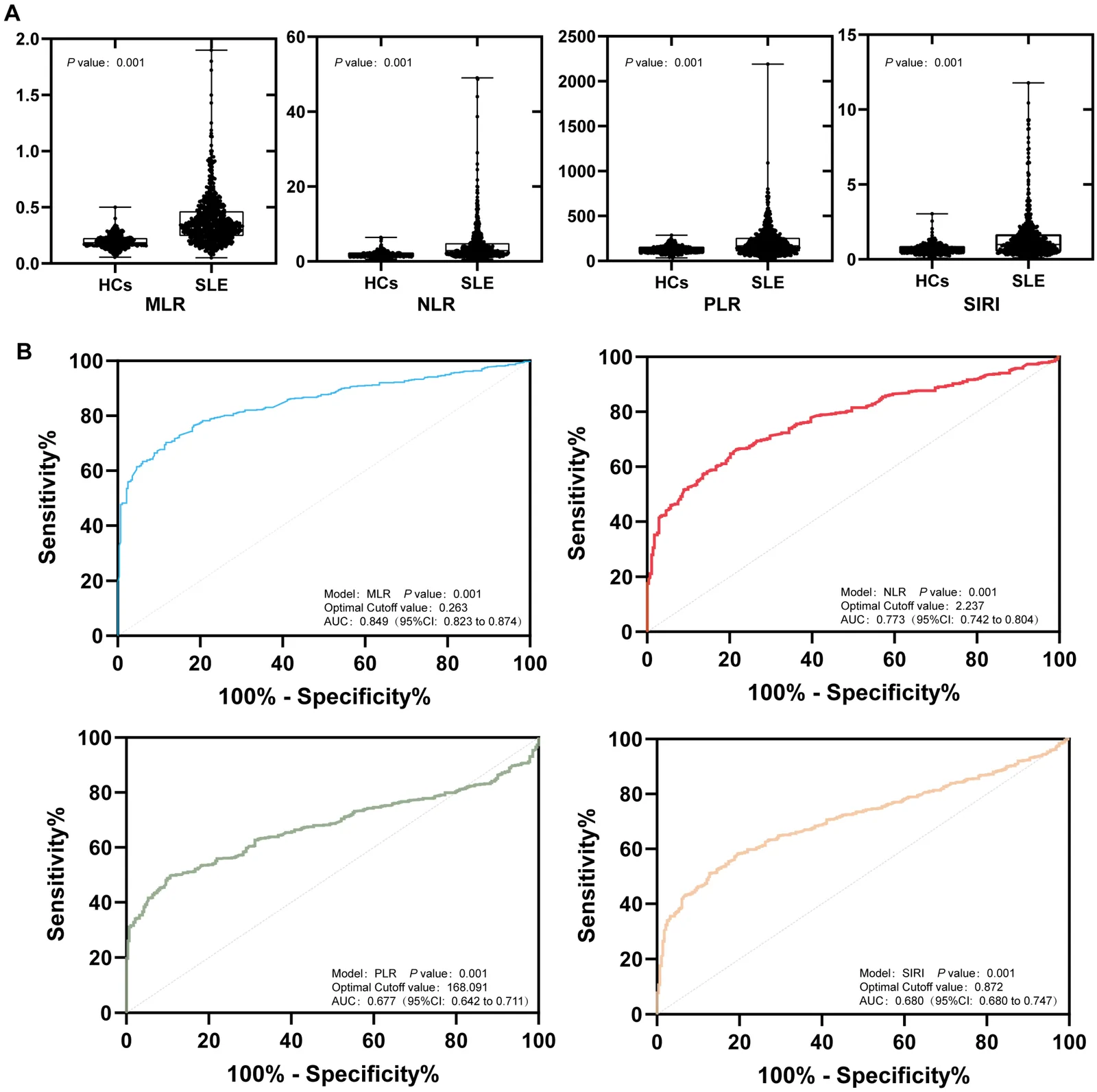
Exploratory comparison of systemic inflammatory response biomarkers between patients with SLE and HCs. (**A**) Comparison of MLR, NLR, PLR, and SIRI between SLE patients and HCs. (**B**) ROC curves of MLR, NLR, PLR, and SIRI comparing SLE patients with HCs. This analysis was performed to characterize inflammatory profiles and was not intended to establish diagnostic use for SLE.

A total of 579 patients with SLE and 282 healthy donors were included in this study. Table 1 shows the exploratory descriptive comparison between SLE patients and healthy controls. No significant differences were observed between the two groups in terms of age or sex. The counts of WBC, monocytes, lymphocytes, platelets and Hb were lower in patients with SLE compared with HCs. Compared with the healthy controls, SIRI, NLR, PLR and MLR were higher in patients with SLE (Figure 1A), indicating a higher systemic inflammatory burden in SLE. This comparison was intended to characterize inflammatory profiles rather than to establish CBC-derived indices as diagnostic tools for SLE.

In an exploratory ROC analysis comparing SLE patients with HCs, MLR (cutoff: 0.263, Sensitivity: 0.703, Specificity: 0.883), NLR (cutoff: 2.237, Sensitivity: 0.663, Specificity: 0.784), SIRI (cutoff: 0.872, Sensitivity 0.582, Specificity 0.805) and PLR (cutoff: 168.091, Sensitivity: 0.497, Specificity: 0.894) showed discriminatory ability between the two groups (Figure 1B). These findings should be interpreted as descriptive rather than diagnostic.

### 3.2. Relationship Between Systemic Inflammation Response Biomarkers and SLEDAI-2K in SLE

**Figure 2:**
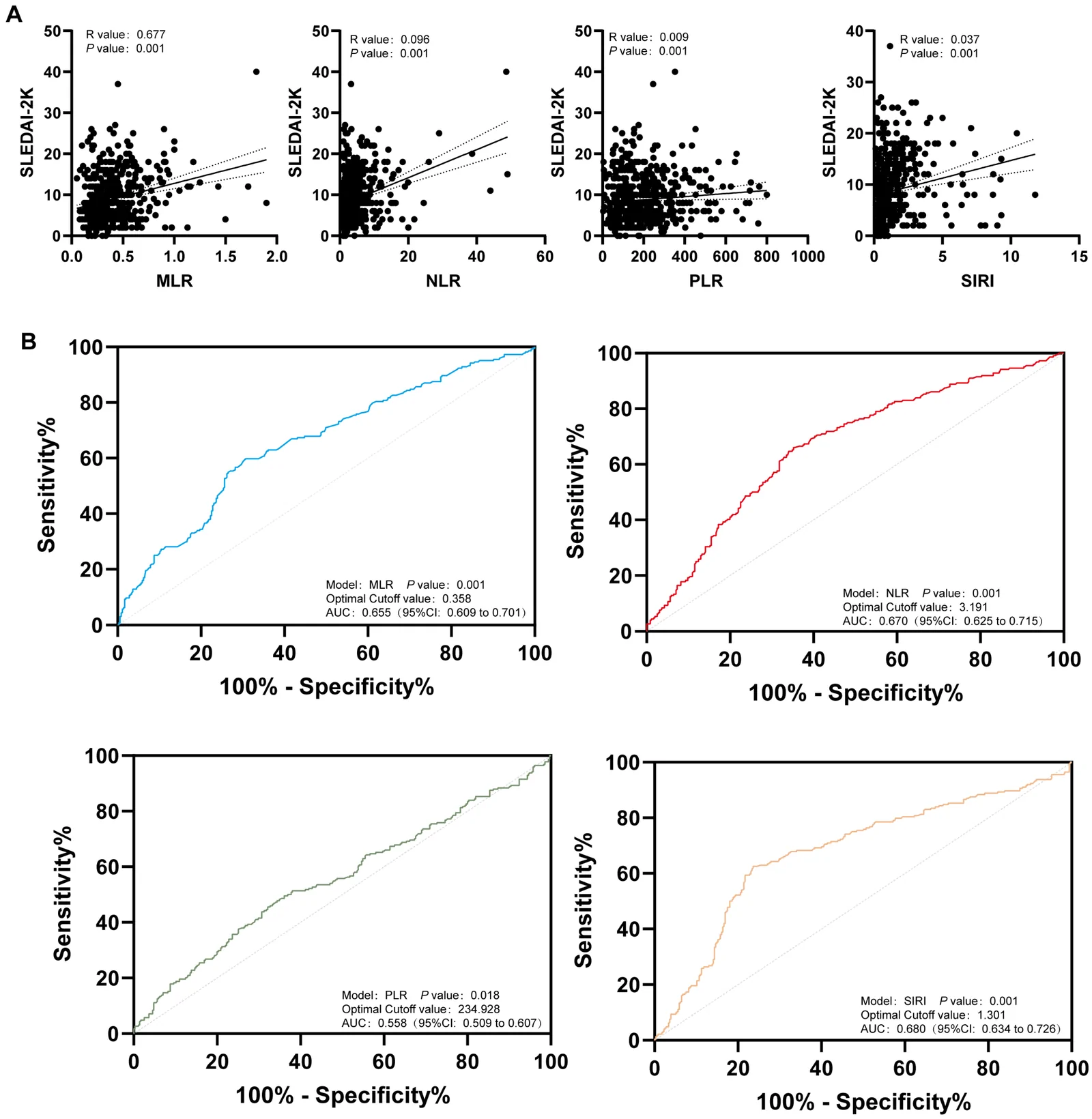
Association of MLR, NLR, PLR, and SIRI with SLE disease activity. (**A**) Spearman correlations between MLR, NLR, PLR, SIRI, and SLEDAI-2K in patients with SLE. (**B**) ROC curves of MLR, NLR, PLR, and SIRI for exploratory discrimination of high disease activity from low-to-moderate disease activity.

Given the elevated CBC-derived inflammatory biomarkers in SLE patients, we explored whether these indices were associated with SLE disease activity assessed by SLEDAI-2K. Spearman correlation analysis showed that SIRI, NLR, PLR, and MLR were all positively correlated with SLEDAI-2K (Figure 2A), suggesting that these biomarkers may reflect systemic inflammatory burden to some extent. Patients were then divided into low-to-moderate disease activity and high disease activity groups according to SLEDAI-2K, with high disease activity defined as SLEDAI-2K ≥ 10. Table 2 shows the comparison between the two groups.

Exploratory ROC analysis indicated that MLR (cutoff: 0.358, sensitivity: 0.548, specificity: 0.638), NLR (cutoff: 3.191, sensitivity: 0.594, specificity: 0.581), SIRI (cutoff: 1.301, sensitivity: 0.490, specificity: 0.652), and PLR (cutoff: 243.928, sensitivity: 0.381, specificity: 0.733) had modest ability to discriminate high disease activity from low-to-moderate disease activity (Figure 2B).

These findings suggest that CBC-derived inflammatory indices may provide adjunctive information for rapid assessment of SLE disease activity, but they should not replace established disease activity indices or organ-specific clinical evaluation.

### 3.3. Systemic Inflammation Response Biomarkers and Immunological Findings in SLE

**Figure 3:**
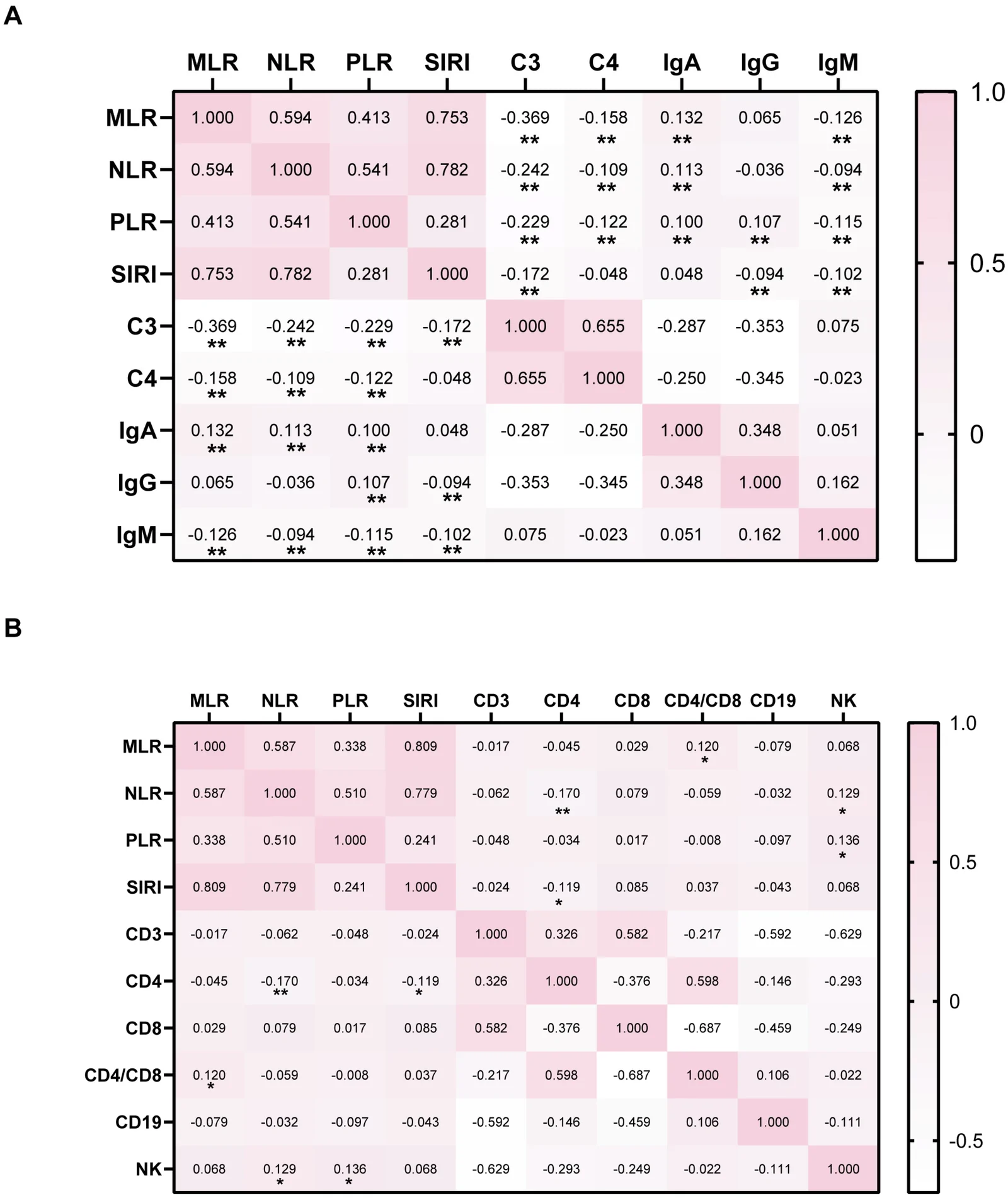
Correlations between CBC-derived inflammatory biomarkers and immunological parameters in SLE. (**A**) Correlations of MLR, NLR, PLR, and SIRI with C3, C4, IgA, IgG, and IgM in all SLE patients (*n* = 579). (**B**) Correlations of MLR, NLR, PLR, and SIRI with CD3^+^ T cells, CD4^+^ T cells, CD8^+^ T cells, the CD4^+^/CD8^+^ T-cell ratio, CD19^+^ B cells, and NK cells in patients from Nantong First People’s Hospital (*n* = 365). Spearman correlation analysis was used. * *p* < 0.05, ** *p* < 0.01.

We further analyzed the systemic inflammation response biomarkers and immunologic data of patients with SLE. In immunological findings, to evaluate the role of the systemic inflammation index in the disease activity of SLE, we compared the correlations between the systemic inflammation score and different immune indicators in SLE patients (Figure 3A). The heatmap results showed that MLR was correlated with C3 (R value: −0.369, *p* value: <0.001), C4 (R value: −0.158, *p* value: <0.001), IgA (R value: −0.132, *p* value: <0.001), IgM (R value: −0.126, *p* value: 0.006), NLR was correlated with C3 (R value: −0.242, *p* value: <0.001), C4 (R value: −0.109, *p* value: <0.001), IgA (R value:0.113, *p* value: <0.001), and IgM (R value: −0.094, *p* value: 0.006), PLR was correlated with C3 (R value: −0.229, *p* value: <0.001), C4 (R value: −0.122, *p* value: <0.001), IgA (R value: −0.100, *p* value: 0.003), IgG (R value: −0.107, *p* value: 0.002), and IgM (R value: −0.115, *p* value: <0.001), and SIRI was correlated with C3 (R value: −0.172, *p* value: <0.001), IgG (R value: −0.094, *p* value: 0.006), and IgM (R value: −0.102, *p* value: 0.003).

In the immunologic analysis, we further examined the associations between levels of CD3^+^ T cells, CD4^+^ T cells, CD8^+^ T cells, the CD4^+^/CD8^+^ T-cell ratio, CD19^+^ B cells, and NK cells, and systemic inflammation response biomarkers (Figure 3B). We found that MLR with CD4^+^ T cells/CD8^+^ T cells (R value: 0.120, *p* value: 0.003), NLR with CD4 (R value: −0.170, *p* value: 0.003) and NK (R value: 0.129, *p* value: 0.003), PLR with NK (R value: 0.136, *p* value: 0.003), SIRI with CD4 (R value: −0.119, *p* value: 0.003) were correlated. This result indicates that the high level of systemic inflamm ation response biomarkers may reflect an imbalance in immune cells.

### 3.4. Neuropsychiatric Involvement and Discriminatory Performance of Inflammatory Biomarkers

**Figure 4:**
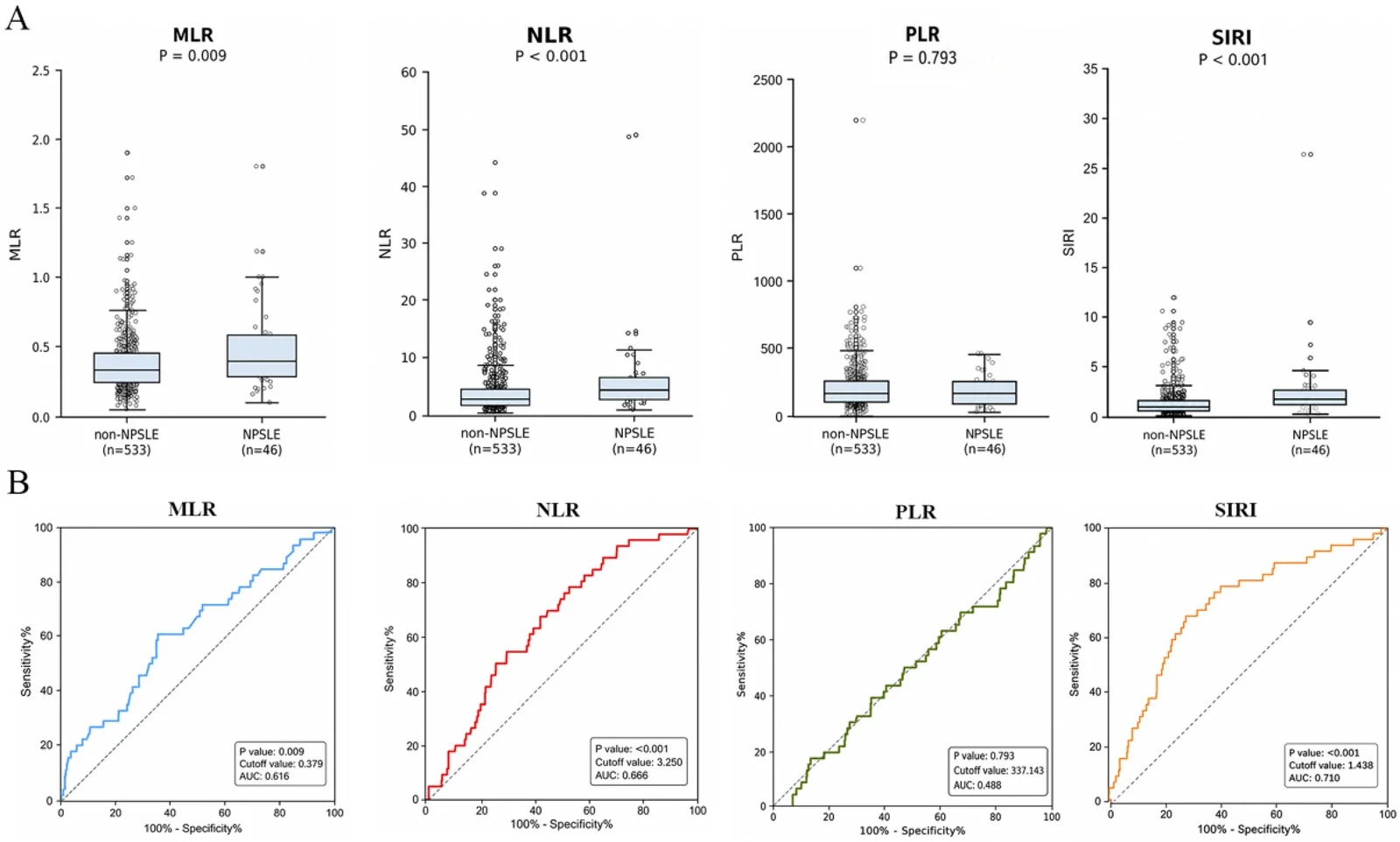
Comparisons and exploratory discriminatory performance of CBC-derived inflammatory biomarkers for neuropsychiatric involvement in SLE. (**A**) Box-and-whisker plots comparing MLR, NLR, PLR, and SIRI between patients with non-NPSLE (*n* = 533) and NPSLE (*n* = 46). The center line indicates the median, the box indicates the interquartile range, and the whiskers indicate the data range. Each dot represents one patient. *p* values were calculated using the Mann–Whitney U test. (**B**) ROC curves of MLR, NLR, PLR, and SIRI for exploratory discrimination between NPSLE and non-NPSLE. The AUC, optimal cutoff value, and *p* value are shown in each panel.

Among the 579 SLE patients, 46 were classified as having NPSLE and 533 as having non-NPSLE. Table 3 shows the comparison between NPSLE patients and Non-NPSLE patients. As shown in Figure 4A, patients with NPSLE had significantly higher MLR, NLR, and SIRI levels than those with non-NPSLE (MLR, *p* = 0.008; NLR, *p* < 0.001; SIRI, *p* < 0.001), whereas PLR did not differ significantly between the two groups (*p* = 0.941).

ROC curve analysis was further used to explore whether these biomarkers could provide auxiliary information for distinguishing NPSLE from non-NPSLE (Figure 4B). The AUCs were 0.616 for MLR, 0.666 for NLR, 0.488 for PLR, and 0.710 for SIRI. The corresponding optimal cutoff values were 0.379, 3.250, 337.140, and 1.438, respectively. Among the four biomarkers, SIRI showed the highest exploratory discriminatory ability for NPSLE, followed by NLR and MLR, whereas PLR showed limited discriminatory value. Given the limited number and heterogeneity of NPSLE cases, these results should be interpreted cautiously.

### 3.5. Adjusted Associations Between Inflammatory Biomarkers and SLEDAI-2K Score

**Figure 5:**
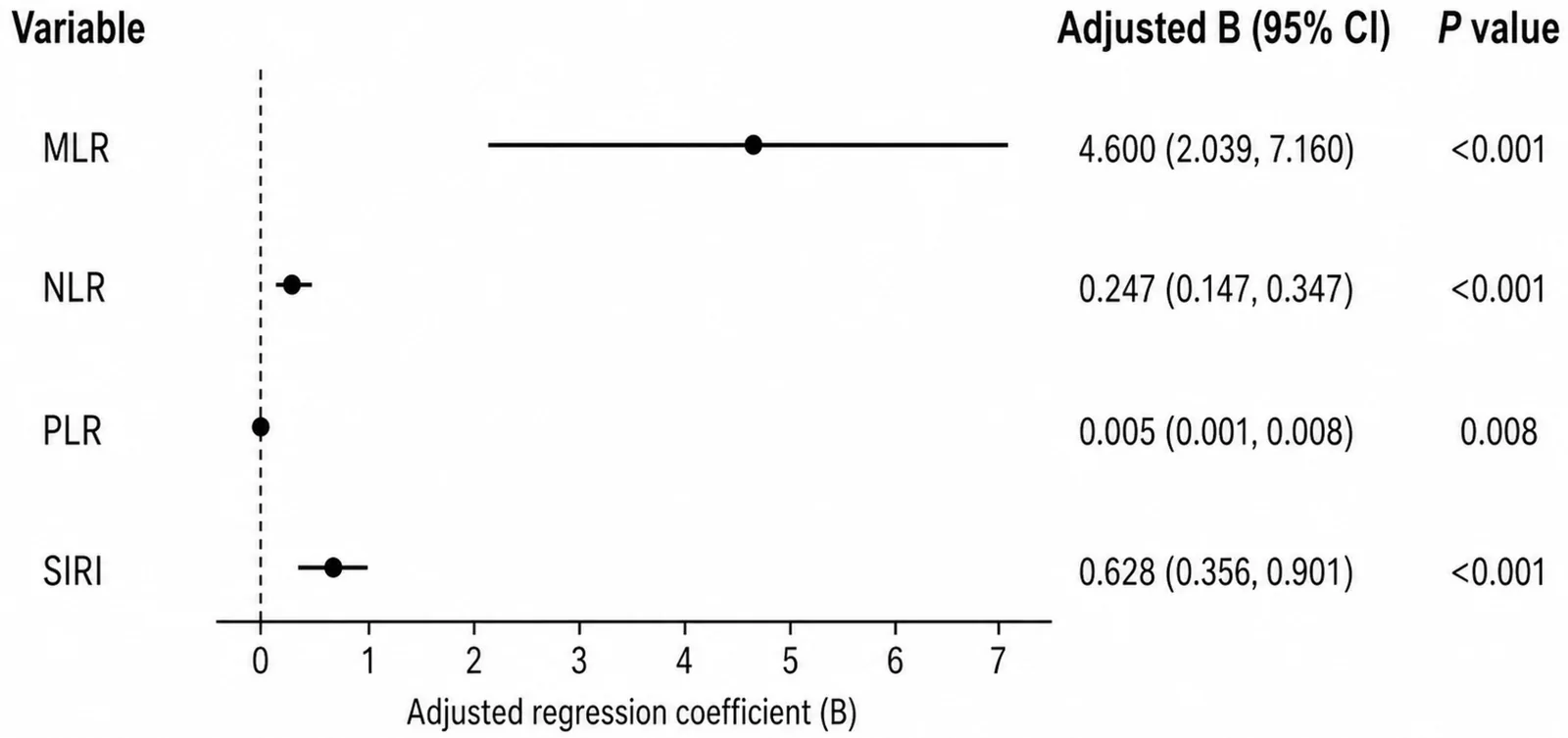
Adjusted associations of CBC-derived inflammatory biomarkers with SLE disease activity. Summary forest plot showing the adjusted regression coefficients of MLR, NLR, PLR, and SIRI for the SLEDAI-2K score. Four separate multivariable linear regression models were constructed for MLR, NLR, PLR, and SIRI, respectively. Each model was adjusted for sex, age, disease duration, hemoglobin, ESR, and CRP. Center-adjusted linear regression, logistic regression for high disease activity, and non-hematologic SLEDAI-2K sensitivity analyses are provided in Supplementary Tables S6, S7, and S11. Dots represent adjusted regression coefficients, horizontal lines indicate 95% CIs, and the dashed vertical line indicates a regression coefficient of 0. Complete-case sample size for these analyses was *n* = 555.

In multivariable linear regression analyses adjusted for sex, age, disease duration, hemoglobin, ESR, and CRP, all four CBC-derived inflammatory biomarkers were positively associated with SLEDAI-2K score. As summarized in Figure 5, the adjusted regression coefficient for MLR was 4.600 (95% CI, 2.039–7.160; *p* < 0.001), for NLR was 0.247 (95% CI, 0.147–0.347; *p* < 0.001), for PLR was 0.005 (95% CI, 0.001–0.008; *p* = 0.008), and for SIRI was 0.628 (95% CI, 0.356–0.901; *p* < 0.001). After additional adjustment for the study center, the associations remained statistically significant (Supplementary Table S6). Sensitivity analyses using high disease activity as a binary outcome and non-hematologic SLEDAI-2K as an alternative outcome yielded generally consistent results (Supplementary Tables S7 and S11).

These findings indicate that higher levels of MLR, NLR, PLR, and SIRI were associated with increased SLE disease activity. Because these indices share overlapping blood cell components and may be influenced by treatment exposure or hematological manifestations, they should be interpreted as adjunctive indicators rather than independent mechanistic biomarkers.

## 4. Discussion

Systemic lupus erythematosus (SLE) is a prototypical chronic autoimmune disease characterized by high clinical heterogeneity and an unpredictable disease course [11,12]. Accurate assessment of disease activity is paramount for tailoring therapeutic strategies and improving patient prognosis [13,14]. While the SLEDAI-2K index is the clinical standard for disease activity assessment, it relies on clinical and laboratory parameters that may not always be available in real time [15,16]. Consequently, accessible and cost-effective adjunctive biomarkers derived from routine hematological parameters may be useful for rapid clinical assessment. In this multicenter study, systemic inflammatory response biomarkers, including NLR, MLR, PLR, and SIRI, were elevated in SLE patients compared with healthy controls. This comparison was descriptive and should not be interpreted as supporting the use of these indices for SLE diagnosis. Instead, the main clinical focus of this study was disease activity assessment and exploratory NPSLE risk stratification.

MLR showed a strong association with SLEDAI-2K score, and NLR, PLR, and SIRI were also positively associated with disease activity. These findings suggest that CBC-derived indices may reflect systemic inflammatory burden in SLE. Consistent with previous studies, these markers were also associated with complement consumption and immune cell subset imbalance [17,18]. However, because NLR, MLR, PLR, and SIRI share overlapping blood cell components, they should not be interpreted as completely independent biological signals. Rather, they may provide complementary information that can be integrated with conventional disease activity assessment.

Organ-specific involvement, particularly neuropsychiatric lupus (NPSLE), remains clinically challenging [19,20]. In this study, SIRI showed modest exploratory discriminatory ability for NPSLE. Nevertheless, NPSLE is a heterogeneous syndrome, and the number of NPSLE cases was limited [21]. Therefore, SIRI should be interpreted only as a potential auxiliary risk-stratification marker rather than a specific or independent biomarker for NPSLE. Detailed NPSLE phenotype distribution and attribution criteria, including the requirement for available objective support from MRI, CSF analysis, neurological examination, or electrophysiological testing, are summarized in Supplementary Tables S9 and S10.

A unique challenge in SLE is systemic cytopenia. Because SLEDAI-2K itself includes hematologic components, the relationship between CBC-derived indices and total SLEDAI-2K may partly reflect overlap with hematological disease activity [22,23]. To address this issue, we performed sensitivity analyses using non-hematologic SLEDAI-2K after subtracting leukopenia and thrombocytopenia items, and the associations remained generally consistent (Supplementary Table S11). These findings suggest that CBC-derived indices may reflect broader inflammatory burden, although residual overlap with hematological abnormalities cannot be fully excluded.

Several confounding factors should be considered. Corticosteroids can induce neutrophilia and lymphopenia, potentially increasing NLR and SIRI independently of intrinsic disease activity. Immunosuppressive and biologic therapies, infection, renal involvement, anemia, leukopenia, and thrombocytopenia may also affect CBC-derived indices. Treatment data were available for patients from Center 1 and were summarized and explored as an available-case sensitivity analysis (Supplementary Tables S2 and S3); however, detailed cumulative corticosteroid dose and recent pulse therapy were not uniformly available across all centers. In addition, residual inter-center heterogeneity related to laboratory platforms, patient composition, and treatment patterns may still exist despite center adjustment (Supplementary Tables S4–S8). Future prospective studies with standardized treatment recording and longitudinal sampling are warranted.

In conclusion, CBC-derived inflammatory indices (NLR, MLR, PLR, and SIRI) are simple, rapid, and low-cost adjunctive markers associated with SLE disease activity. These indices may help provide rapid supplementary information for clinical assessment, particularly when interpreted together with conventional disease activity indices, organ-specific evaluation, and treatment status. They should not be used as stand-alone diagnostic tools or substitutes for established clinical assessment frameworks.

## 5. Conclusions

In summary, this multicenter retrospective study demonstrates that CBC-derived inflammatory response biomarkers, specifically MLR, NLR, PLR, and SIRI, are accessible and cost-effective adjunctive indicators associated with disease activity in patients with SLE. SIRI may provide supplementary information for identifying patients at higher risk of neuropsychiatric involvement, but this finding should be interpreted cautiously because of the limited number and heterogeneity of NPSLE cases and the possibility of residual confounding.

These routine hematological parameters may support rapid clinical assessment when interpreted together with SLEDAI-2K, organ-specific evaluation, infection assessment, and treatment status. Future prospective longitudinal studies are warranted to validate these biomarkers and to determine whether they can improve risk stratification or monitoring in routine clinical practice.

## Figures and Tables

**Table 1:** Characteristics of healthy controls and patients with SLE.

Characteristic	Healthy Controls	SLE Patients	*p* Value
Age (years), median (IQR)	40.00 (33.00, 47.00)	41.00 (30.00, 53.00)	0.355
Female Sex, *n*/*N* (%)	258/282 (91.5%)	527/579 (91.0%)	0.820
Duration (months), median (IQR)	-	48.00 (6.00, 120.00)	-
SLEDAI-2K, median (IQR)	-	8.00 (5.00, 12.00)	-
WBC (10^9^/L), median (IQR)	5.80 (4.90, 6.80)	4.53 (3.41, 6.12)	<0.001
Monocytes (10^9^/L), median (IQR)	0.37 (0.30, 0.43)	0.34 (0.23, 0.45)	0.012
Neutrophils (10^9^/L), median (IQR)	3.30 (2.60, 4.00)	2.99 (2.15, 4.50)	0.021
Lymphocytes (10^9^/L), median (IQR)	2.00 (1.70, 2.40)	1.00 (0.70, 1.40)	<0.001
Platelets (10^9^/L), median (IQR)	234.00 (199.00, 268.50)	171.00 (124.50, 224.50)	<0.001
Hb (g/L), median (IQR)	135.00 (127.25, 141.75)	115.00 (101.00, 127.00)	<0.001
MLR, median (IQR)	0.18 (0.16, 0.23)	0.33 (0.25, 0.46)	<0.001
NLR, median (IQR)	1.67 (1.32, 2.16)	2.88 (1.89, 4.86)	<0.001
PLR, median (IQR)	120.56 (96.04, 149.19)	167.14 (108.15, 257.20)	<0.001
SIRI, median (IQR)	0.61 (0.46, 0.82)	1.00 (0.58, 1.62)	<0.001

**Table 2:** Characteristics of SLE patients with low-to-moderate disease activity and high disease activity.

Characteristic	Low-to-Moderate Disease Activity	High Disease Activity	*p* Value
Age (years), median (IQR)	41.00 (31.00, 52.75)	41.00 (29.00, 53.00)	0.574
Female Sex, *n*/*N* (%)	328/355 (92.4%)	199/224 (88.8%)	0.191
Duration (months), median (IQR)	60.00 (10.00, 120.00)	30.00 (3.00, 120.00)	0.005
SLEDAI-2K, median (IQR)	6.00 (4.00, 7.00)	13.00 (11.00, 16.25)	<0.001
WBC (10^9^/L), median (IQR)	4.40 (3.41, 5.77)	4.86 (3.40, 6.50)	0.101
Monocytes (10^9^/L), median (IQR)	0.34 (0.24, 0.44)	0.33 (0.22, 0.49)	0.763
Neutrophils (10^9^/L), median (IQR)	2.70 (2.02, 4.10)	3.60 (2.37, 4.85)	<0.001
Lymphocytes (10^9^/L), median (IQR)	1.10 (0.75, 1.50)	0.90 (0.60, 1.20)	<0.001
Platelets (10^9^/L), median (IQR)	176.00 (131.00, 227.00)	164.00 (107.25, 224.00)	0.068
Hb (g/L), median (IQR)	119.00 (107.00, 130.00)	106.50 (95.00, 123.00)	<0.001
MLR, median (IQR)	0.30 (0.23, 0.40)	0.39 (0.28, 0.55)	<0.001
NLR, median (IQR)	2.40 (1.67, 3.86)	3.79 (2.45, 6.54)	<0.001
PLR, median (IQR)	161.43 (105.88, 240.00)	192.92 (113.18, 289.64)	0.018
SIRI, median (IQR)	0.84 (0.52, 1.20)	1.43 (0.86, 2.07)	<0.001

**Table 3:** Characteristics of SLE patients with NPSLE and non-NPSLE.

Characteristic	Non-NPSLE	NPSLE	*p* Value
Age (years), median (IQR)	41.00 (30.00, 53.00)	41.00 (28.00, 54.00)	0.893
Female Sex, *n*/*N* (%)	485/533 (91.0%)	42/46 (91.3%)	0.679
Duration (months), median (IQR)	48.00 (4.00, 120.00)	30.00 (9.00, 120.00)	0.890
SLEDAI-2K, median (IQR)	7.00 (4.00, 11.00)	17.00 (14.00, 20.00)	<0.001
WBC (10^9^/L), median (IQR)	4.50 (3.40, 6.00)	5.79 (3.70, 9.20)	<0.001
Monocytes (10^9^/L), median (IQR)	0.33 (0.22, 0.44)	0.40 (0.30, 0.51)	0.016
Neutrophils (10^9^/L), median (IQR)	2.91 (2.10, 4.32)	4.20 (2.60, 7.10)	<0.001
Lymphocytes (10^9^/L), median (IQR)	1.00 (0.70, 1.40)	1.00 (0.60, 1.30)	0.498
Platelets (10^9^/L), median (IQR)	173.00 (128.00, 224.00)	155.00 (100.00, 230.00)	0.378
Hb (g/L), median (IQR)	115.00 (102.00, 128.00)	103.00 (95.00, 122.00)	0.013
MLR, median (IQR)	0.33 (0.24, 0.46)	0.39 (0.28, 0.59)	0.008
NLR, median (IQR)	2.84 (1.83, 4.60)	4.70 (2.66, 6.75)	<0.001
PLR, median (IQR)	166.07 (108.59, 260.83)	175.00 (92.00, 254.86)	0.941
SIRI, median (IQR)	0.96 (0.57, 1.55)	1.75 (1.17, 2.68)	<0.001

## Data Availability

The original contributions presented in this study are included in the article/Supplementary Materials. Further inquiries can be directed to the corresponding authors.

## Supplementary Materials

The following supporting information can be downloaded at: https://www.mdpi.com/article/10.3390/diagnostics16121944/s1, Table S1. Clinical and laboratory characteristics according to SLE disease activity, Table S2. Treatment characteristics according to SLE disease activity in Center 1, Table S3. Treatment characteristics according to NPSLE status in Center 1, Table S4. Distribution of patients across study centers, Table S5. Center-stratified clinical and laboratory characteristics, Table S6. Associations between CBC-derived inflammatory biomarkers and total SLEDAI-2K before and after center adjustment, Table S7. Logistic regression analyses for high disease activity before and after center adjustment, Table S8. Center 1 sensitivity analysis comparing healthy controls and SLE patients, Table S9. Distribution of NPSLE phenotypes, Table S10. NPSLE attribution criteria and exclusion of mimics, Table S11. Sensitivity analyses using non-hematologic SLEDAI-2K, Table S12. Exploratory organ-specific activity analyses, Table S13. Abbreviations.

## Abbreviations

SLE	systemic lupus erythematosus
NPSLE	neuropsychiatric systemic lupus erythematosus
SLEDAI-2K	SLE Disease Activity Index 2000
ACR	American College of Rheumatology
NLR	neutrophil to lymphocyte ratio
MLR	monocyte to lymphocyte ratio
PLR	Platelet to lymphocyte ratio
SIRI	systemic inflammation response index
RA	rheumatoid arthritis
AS	ankylosing spondylitis
ROC	receiver operating characteristic
GC	glucocorticoids
WBC	white blood cell count
CSF	cerebrospinal fluid
ANOVA	one-way analysis of variance
CBC	complete blood counts
NETs	neutrophil extracellular traps
