# Supplementary material for: Association Between Systemic Inflammatory Response Biomarkers and Disease Activity in Systemic Lupus Erythematosus: A Multi-Center Retrospective Study
Source: Diagnostics (Basel). 2026 Jun 22;16(12):1944. doi: 10.3390/diagnostics16121944 (PMC13298265; doi:10.3390/diagnostics16121944)
Supplement: Supplementary file 1 [file diagnostics-16-01944-s001.zip › diagnostics-4331976-supplementary.pdf]

Supplementary Materials

Association between systemic inflammatory response biomarkers and disease activity in systemic lupus erythematosus: a multi-center retrospective study

General note. Continuous variables are presented as median (interquartile range [IQR]) unless otherwise specified. Categorical variables are presented as n/N (%). Treatment analyses were available-case analyses restricted to Center 1 because treatment data were available for Center 1 only. Center-adjusted models included study center as a categorical fixed-effect covariate. A non-hematologic SLEDAI-2K score was calculated by subtracting hematologic components from the total SLEDAI-2K score.

Table S1. Clinical and laboratory characteristics according to SLE disease activity.

Variable	Low-to-moderate disease activity (n=355)	High disease activity (n=224)	P value	Available n (low/high)
Age, years	41.00 (31.00, 52.75)	41.00 (29.00, 53.00)	0.574	354/224
Female sex	328/355 (92.4)	199/224 (88.8)	0.191	355/224
Disease duration, months	60.00 (10.00, 120.00)	30.00 (3.00, 120.00)	0.005	349/221
SLEDAI-2K score	6.00 (4.00, 7.00)	13.00 (11.00, 16.25)	<0.001	355/224
Non-hematologic SLEDAI-2K score	5.00 (4.00, 6.00)	13.00 (11.00, 16.00)	<0.001	355/224
ESR, mm/h	24.00 (12.00, 42.00)	34.00 (14.00, 59.25)	0.001	354/224
CRP, mg/L	2.13 (0.63, 7.96)	2.90 (0.57, 10.51)	0.311	345/220
C3, g/L	0.70 (0.52, 0.90)	0.56 (0.38, 0.75)	<0.001	355/224
C4, g/L	0.14 (0.09, 0.21)	0.11 (0.06, 0.17)	<0.001	355/224
24-h urinary protein, g/24h	0.15 (0.09, 0.23)	0.29 (0.15, 1.48)	<0.001	355/224
Renal involvement	96/355 (27.0)	133/224 (59.4)	<0.001	355/224
Mucocutaneous involvement	137/355 (38.6)	112/224 (50.0)	0.009	355/224
Articular involvement	89/355 (25.1)	85/224 (37.9)	0.001	355/224
Hematologic involvement	102/355 (28.7)	92/224 (41.1)	0.003	355/224
Neuropsychiatric involvement	3/355 (0.8)	43/224 (19.2)	<0.001	355/224
Anti-dsDNA positivity (available qualitative results)	38/104 (36.5)	22/51 (43.1)	0.537	104/51
ANA positivity (available qualitative results)	99/104 (95.2)	81/90 (90.0)	0.265	104/90

Note. Non-hematologic SLEDAI-2K was calculated by excluding hematologic components from the total SLEDAI-2K score. P values were calculated using Mann-Whitney U test, chi-square test, or Fisher exact test as appropriate.

Table S2. Treatment characteristics according to SLE disease activity in Center 1.

Treatment variable	Low-to-moderate disease activity (n=210)	High disease activity (n=155)	P value	Statistical test

Treatment variable	Low-to-moderate disease activity (n=210)	High disease activity (n=155)	P value	Statistical test
Glucocorticoids	179/210 (85.2%)	131/155 (84.5%)	0.849	Chi-square test
Hydroxychloroquine	132/210 (62.9%)	83/155 (53.5%)	0.074	Chi-square test
Conventional immunosuppressants	86/210 (41.0%)	62/155 (40.0%)	0.855	Chi-square test
Methotrexate	6/210 (2.9%)	3/155 (1.9%)	0.738	Fisher exact test
Leflunomide	5/210 (2.4%)	3/155 (1.9%)	1	Fisher exact test
Mycophenolate mofetil	46/210 (21.9%)	41/155 (26.5%)	0.314	Chi-square test
Cyclosporine	6/210 (2.9%)	4/155 (2.6%)	1	Fisher exact test
Tacrolimus	6/210 (2.9%)	1/155 (0.6%)	0.246	Fisher exact test
Azathioprine	3/210 (1.4%)	2/155 (1.3%)	1	Fisher exact test
Iguratimod	11/210 (5.2%)	5/155 (3.2%)	0.353	Chi-square test
Cyclophosphamide	4/210 (1.9%)	6/155 (3.9%)	0.335	Fisher exact test
Biologics	28/210 (13.3%)	33/155 (21.3%)	0.044	Chi-square test
Rituximab	7/210 (3.3%)	11/155 (7.1%)	0.101	Chi-square test
Belimumab	10/210 (4.8%)	5/155 (3.2%)	0.465	Chi-square test
Telitacicept	11/210 (5.2%)	17/155 (11.0%)	0.042	Chi-square test
Tofacitinib	3/210 (1.4%)	1/155 (0.6%)	0.64	Fisher exact test
Any immunomodulatory therapy	184/210 (87.6%)	132/155 (85.2%)	0.496	Chi-square test

Note. Treatment information was available from Center 1 only and was analyzed as an available-case sensitivity analysis. Detailed cumulative corticosteroid dose and recent pulse therapy were not uniformly available.

Table S3. Treatment characteristics according to NPSLE status in Center 1.

Treatment variable	non-NPSLE (n=334)	NPSLE (n=31)	P value	Statistical test
Glucocorticoids	285/334 (85.3%)	25/31 (80.6%)	0.44	Fisher exact test
Hydroxychloroquine	201/334 (60.2%)	14/31 (45.2%)	0.104	Chi-square test
Conventional immunosuppressants	139/334 (41.6%)	9/31 (29.0%)	0.172	Chi-square test
Methotrexate	9/334 (2.7%)	0/31 (0.0%)	1	Fisher exact test
Leflunomide	8/334 (2.4%)	0/31 (0.0%)	1	Fisher exact test
Mycophenolate mofetil	81/334 (24.3%)	6/31 (19.4%)	0.54	Chi-square test
Cyclosporine	8/334 (2.4%)	2/31 (6.5%)	0.205	Fisher exact test
Tacrolimus	7/334 (2.1%)	0/31 (0.0%)	1	Fisher exact test
Azathioprine	4/334 (1.2%)	1/31 (3.2%)	0.36	Fisher exact test
Iguratimod	16/334 (4.8%)	0/31 (0.0%)	0.38	Fisher exact test

Treatment variable	non-NPSLE (n=334)	NPSLE (n=31)	P value	Statistical test
Cyclophosphamide	10/334 (3.0%)	0/31 (0.0%)	1	Fisher exact test
Biologics	53/334 (15.9%)	8/31 (25.8%)	0.156	Chi-square test
Rituximab	13/334 (3.9%)	5/31 (16.1%)	0.012	Fisher exact test
Belimumab	13/334 (3.9%)	2/31 (6.5%)	0.37	Fisher exact test
Telitacicept	27/334 (8.1%)	1/31 (3.2%)	0.493	Fisher exact test
Tofacitinib	4/334 (1.2%)	0/31 (0.0%)	1	Fisher exact test
Any immunomodulatory therapy	291/334 (87.1%)	25/31 (80.6%)	0.281	Fisher exact test

Note. Treatment information was available from Center 1 only. Because the number of NPSLE cases was limited, these analyses should be interpreted cautiously.

Table S4. Distribution of patients across study centers.

Study center	Total SLE patients, n	Low-to-moderate activity, n/N (%)	High disease activity, n/N (%)	non-NPSLE, n/N (%)	NPSLE, n/N (%)
Center 1	365	210/365 (57.5)	155/365 (42.5)	334/365 (91.5)	31/365 (8.5)
Center 2	74	46/74 (62.2)	28/74 (37.8)	62/74 (83.8)	12/74 (16.2)
Center 3	72	49/72 (68.1)	23/72 (31.9)	72/72 (100.0)	0/72 (0.0)
Center 4	42	31/42 (73.8)	11/42 (26.2)	39/42 (92.9)	3/42 (7.1)
Center 5	26	19/26 (73.1)	7/26 (26.9)	26/26 (100.0)	0/26 (0.0)
Total	579	355/579 (61.3)	224/579 (38.7)	533/579 (92.1)	46/579 (7.9)

Note. Percentages are calculated within each center unless otherwise indicated. Pearson chi-square P values: center distribution across disease activity groups, P=0.096; center distribution across NPSLE status, P=0.004.

Table S5. Center-stratified clinical and laboratory characteristics.

Variable	Center 1	Center 2	Center 3	Center 4	Center 5	P value
Total patients, n	365	74	72	42	26	
Female sex, n/N (%)	330/365 (90.4)	68/74 (91.9)	67/72 (93.1)	39/42 (92.9)	23/26 (88.5)	0.913
High disease activity, n/N (%)	155/365 (42.5)	28/74 (37.8)	23/72 (31.9)	11/42 (26.2)	7/26 (26.9)	0.096
NPSLE, n/N (%)	31/365 (8.5)	12/74 (16.2)	0/72 (0.0)	3/42 (7.1)	0/26 (0.0)	0.004
Age, years	43.0 (30.0, 55.0)	39.0 (30.0, 50.0)	35.0 (28.0, 41.5)	46.0 (40.0, 51.8)	46.5 (32.2, 54.8)	<0.001
Disease duration, months	48.0 (4.2, 120.0)	36.0 (6.0, 84.0)	49.0 (12.0, 97.0)	72.0 (24.0, 120.0)	84.0 (24.0, 120.0)	0.246
SLEDAI-2K score	8.0 (5.0, 12.0)	7.5 (5.0, 12.0)	6.0 (4.0, 10.0)	6.0 (5.0, 9.5)	7.0 (4.2, 9.5)	0.093
Hemoglobin, g/L	113.0 (100.0, 125.0)	121.5 (105.5, 131.0)	117.0 (99.8, 125.2)	117.0 (96.0, 134.8)	117.5 (103.2, 126.5)	0.169
ESR, mm/h	25.0 (11.0, 48.0)	29.0 (13.0, 46.0)	33.5 (19.0, 49.2)	31.5 (13.0, 60.8)	23.0 (13.5, 35.0)	0.081
CRP, mg/L	1.23 (0.15, 7.80)	4.67 (1.33, 10.77)	5.05 (1.52, 8.56)	5.83 (2.45, 13.09)	2.71 (1.94, 4.27)	<0.001

Variable	Center 1	Center 2	Center 3	Center 4	Center 5	P value
C3, g/L	0.70 (0.50, 0.90)	0.57 (0.36, 0.77)	0.61 (0.43, 0.79)	0.56 (0.47, 0.68)	0.56 (0.30, 0.67)	<0.001
C4, g/L	0.15 (0.09, 0.21)	0.09 (0.04, 0.15)	0.12 (0.06, 0.19)	0.11 (0.08, 0.14)	0.10 (0.06, 0.15)	<0.001
MLR	0.333 (0.248, 0.475)	0.294 (0.198, 0.396)	0.334 (0.254, 0.471)	0.328 (0.278, 0.399)	0.333 (0.255, 0.418)	0.266
NLR	3.154 (1.900, 5.571)	2.931 (2.134, 4.783)	2.507 (1.640, 3.920)	2.155 (1.754, 3.036)	2.572 (1.870, 4.183)	0.002
PLR	173.33 (112.50, 270.00)	133.76 (81.54, 201.56)	192.14 (118.05, 282.47)	150.94 (128.91, 214.24)	143.13 (75.06, 233.30)	0.012
SIRI	1.080 (0.648, 1.823)	0.905 (0.545, 1.589)	0.899 (0.507, 1.402)	0.856 (0.504, 1.267)	0.869 (0.652, 1.196)	0.012

Note. Continuous variables are presented as median (IQR); categorical variables are presented as n/N (%). P values were calculated using Kruskal-Wallis test for continuous variables and chi-square test or Fisher exact test for categorical variables.

Table S6. Associations between CBC-derived inflammatory biomarkers and total SLEDAI-2K before and after center adjustment.

Biomarker	Model	N	B	95% CI	P value	R^2
MLR	Crude	579	6.398	4.456, 8.339	<0.001	0.068
MLR	Adjusted without center	555	6.133	4.231, 8.035	<0.001	0.154
MLR	Adjusted with center	555	5.926	4.027, 7.824	<0.001	0.171
NLR	Crude	579	0.342	0.256, 0.428	<0.001	0.096
NLR	Adjusted without center	555	0.317	0.233, 0.402	<0.001	0.174
NLR	Adjusted with center	555	0.301	0.216, 0.386	<0.001	0.185
PLR	Crude	579	0.0052	0.0025, 0.0080	<0.001	0.023
PLR	Adjusted without center	555	0.0044	0.0016, 0.0071	0.002	0.108
PLR	Adjusted with center	555	0.0041	0.0014, 0.0069	0.003	0.127
SIRI	Crude	579	0.868	0.629, 1.106	<0.001	0.081
SIRI	Adjusted without center	555	0.883	0.650, 1.116	<0.001	0.176
SIRI	Adjusted with center	555	0.844	0.609, 1.079	<0.001	0.188

Note. Adjusted models included age, sex, disease duration, hemoglobin, ESR, and CRP. Center-adjusted models additionally included study center as a categorical fixed-effect covariate. Four separate models were fitted for MLR, NLR, PLR, and SIRI.

Table S7. Logistic regression analyses for high disease activity before and after center adjustment.

Biomarker	Model	N	OR	95% CI	P value
MLR	Crude	579	10.839	4.505, 26.083	<0.001
MLR	Adjusted without center	555	11.552	4.598, 29.025	<0.001
MLR	Adjusted with center	555	10.834	4.282, 27.413	<0.001
NLR	Crude	579	1.118	1.066, 1.173	<0.001

Biomarker	Model	N	OR	95% CI	P value
NLR	Adjusted without center	555	1.105	1.052, 1.159	<0.001
NLR	Adjusted with center	555	1.096	1.044, 1.150	<0.001
PLR	Crude	579	1.002	1.001, 1.003	0.003
PLR	Adjusted without center	555	1.001	1.000, 1.003	0.017
PLR	Adjusted with center	555	1.001	1.000, 1.003	0.025
SIRI	Crude	579	1.295	1.143, 1.467	<0.001
SIRI	Adjusted without center	555	1.333	1.170, 1.518	<0.001
SIRI	Adjusted with center	555	1.307	1.147, 1.491	<0.001

Note. High disease activity was defined as SLEDAI-2K >= 10. Adjusted models included age, sex, disease duration, hemoglobin, ESR, and CRP. Center-adjusted models additionally included study center as a categorical fixed-effect covariate.

Table S8. Center 1 sensitivity analysis comparing healthy controls and SLE patients.

Variable	Healthy controls, Center 1 (n=282)	SLE patients, Center 1 (n=365)	P value
Female sex, n/N (%)	258/282 (91.5)	330/365 (90.4)	0.738
Age, years	40.0 (33.0, 47.0)	43.0 (30.0, 55.0)	0.082
WBC, $\times 10^9$/L	5.80 (4.90, 6.80)	4.80 (3.50, 6.50)	<0.001
Monocytes, $\times 10^9$/L	0.37 (0.30, 0.43)	0.34 (0.24, 0.47)	0.032
Neutrophils, $\times 10^9$/L	3.30 (2.60, 4.00)	3.20 (2.30, 4.70)	0.955
Lymphocytes, $\times 10^9$/L	2.00 (1.60, 2.30)	1.00 (0.70, 1.40)	<0.001
Hemoglobin, g/L	135.0 (127.2, 141.8)	113.0 (100.0, 125.0)	<0.001
Platelets, $\times 10^9$/L	234.0 (199.0, 268.5)	171.0 (125.0, 224.0)	<0.001
MLR	0.185 (0.155, 0.225)	0.333 (0.248, 0.475)	<0.001
NLR	1.667 (1.319, 2.164)	3.154 (1.900, 5.571)	<0.001
PLR	120.56 (96.04, 149.19)	173.33 (112.50, 270.00)	<0.001
SIRI	0.609 (0.459, 0.824)	1.080 (0.648, 1.823)	<0.001

Note. Healthy controls were all from Center 1. This sensitivity analysis compared Center 1 SLE patients with Center 1 healthy controls to reduce center-source confounding in the descriptive SLE versus control comparison.

Table S9. Distribution of NPSLE phenotypes.

NPSLE phenotype	n/N (%)
Seizure disorder	6/46 (13.0)
Psychosis/psychiatric manifestation	8/46 (17.4)
Cerebrovascular disease	21/46 (45.7)

NPSLE phenotype	n/N (%)
Lupus headache	17/46 (37.0)
Peripheral neuropathy	12/46 (26.1)
Two or more neuropsychiatric manifestations	16/46 (34.8)

Note. Patients could have more than one neuropsychiatric manifestation; therefore, percentages may sum to more than 100%.

Table S10. NPSLE attribution criteria and exclusion of mimics.

Item	Description
Classification framework	1999 ACR nomenclature for neuropsychiatric SLE syndromes, together with 2019 EULAR/ACR SLE classification criteria.
Objective support	Neurological examination and available ancillary evidence, including MRI, CSF analysis, or electrophysiological testing where available.
Exclusion of mimics	Cases primarily attributable to active infection, metabolic disturbance, treatment-related complications, primary neurological diseases, intracranial mass lesions, or pre-existing psychiatric disorders unrelated to SLE were excluded.
Interpretation	NPSLE analyses were exploratory because of limited events and phenotype heterogeneity.

Note. The NPSLE analysis was exploratory because of the limited number of NPSLE cases and phenotype heterogeneity.

Table S11. Sensitivity analyses using non-hematologic SLEDAI-2K.

Biomarker	Outcome	N	Adjusted B	95% CI	P value	R^2
MLR	Total SLEDAI-2K	555	5.926	4.027, 7.824	<0.001	0.171
MLR	Non-hematologic SLEDAI-2K	555	5.742	3.841, 7.643	<0.001	0.161
NLR	Total SLEDAI-2K	555	0.301	0.216, 0.386	<0.001	0.185
NLR	Non-hematologic SLEDAI-2K	555	0.305	0.220, 0.391	<0.001	0.181
PLR	Total SLEDAI-2K	555	0.004	0.001, 0.007	0.003	0.127
PLR	Non-hematologic SLEDAI-2K	555	0.005	0.002, 0.007	<0.001	0.125
SIRI	Total SLEDAI-2K	555	0.844	0.609, 1.079	<0.001	0.188
SIRI	Non-hematologic SLEDAI-2K	555	0.877	0.643, 1.111	<0.001	0.188

Note. Models included age, sex, disease duration, hemoglobin, ESR, CRP, and study center. Non-hematologic SLEDAI-2K was calculated by excluding hematologic components from the total SLEDAI-2K score.

Table S12. Exploratory organ-specific activity analyses.

Outcome	Biomarker	N	Adjusted OR	95% CI	P value
Renal involvement	MLR	555	1.915	0.884, 4.147	0.099
Renal involvement	NLR	555	1.055	1.015, 1.097	0.007
Renal involvement	PLR	555	1	0.999, 1.002	0.397
Renal involvement	SIRI	555	1.087	0.981, 1.204	0.111
Neuropsychiatric involvement	MLR	555	1.23	0.282, 5.357	0.783
Neuropsychiatric involvement	NLR	555	0.998	0.939, 1.062	0.96
Neuropsychiatric involvement	PLR	555	NE	NE	NE
Neuropsychiatric involvement	SIRI	555	1.131	0.918, 1.394	0.247
Mucocutaneous involvement	MLR	555	1.219	0.561, 2.650	0.616
Mucocutaneous involvement	NLR	555	0.979	0.941, 1.018	0.292
Mucocutaneous involvement	PLR	555	1	0.999, 1.002	0.396
Mucocutaneous involvement	SIRI	555	0.955	0.858, 1.063	0.404
Articular involvement	MLR	555	0.717	0.301, 1.706	0.452
Articular involvement	NLR	555	0.987	0.948, 1.028	0.528
Articular involvement	PLR	555	1	0.999, 1.001	0.849
Articular involvement	SIRI	555	0.978	0.872, 1.098	0.71

Note. Exploratory logistic regression models adjusted for age, sex, disease duration, hemoglobin, ESR, CRP, and study center. NE indicates that the estimate was not estimable because of sparse data or model instability.

Table S13. Abbreviations.

Abbreviation	Definition
ACR	American College of Rheumatology
ANA	antinuclear antibody
CBC	complete blood count
CI	confidence interval
CRP	C-reactive protein
CSF	cerebrospinal fluid
ESR	erythrocyte sedimentation rate
HCQ	hydroxychloroquine
IQR	interquartile range
MLR	monocyte-to-lymphocyte ratio
NLR	neutrophil-to-lymphocyte ratio

Abbreviation	Definition
NPSLE	neuropsychiatric systemic lupus erythematosus
OR	odds ratio
PLR	platelet-to-lymphocyte ratio
SIRI	systemic inflammation response index
SLE	systemic lupus erythematosus
SLEDAI-2K	Systemic Lupus Erythematosus Disease Activity Index 2000
